# Mesenteric traction syndrome in pigs: A single‐blinded, randomized controlled trial

**DOI:** 10.1002/ame2.12160

**Published:** 2021-03-23

**Authors:** Rune B. Strandby, Jens T. F. Osterkamp, Rikard Ambrus, Amelie Henriksen, Jens P. Goetze, Niels H. Secher, Michael P. Achiam, Lars‐Bo Svendsen

**Affiliations:** ^1^ Department of Surgical Gastroenterology, Rigshospitalet University of Copenhagen Copenhagen Ø Denmark; ^2^ Department of Clinical Biochemistry, Rigshospitalet University of Copenhagen Copenhagen Ø Denmark; ^3^ Department of Anaesthesia, Rigshospitalet University of Copenhagen Copenhagen Ø Denmark

**Keywords:** 6‐keto‐PGF1α, gastric blood flow, hemodynamics, laser speckle contrast imaging, mesenteric traction syndrome, prostacyclin

## Abstract

**Background:**

Mesenteric traction syndrome is commonly observed in patients undergoing upper abdominal surgery and is associated with severe postoperative complications. A triad of hypotension, tachycardia, and facial flushing seems provoked by prostacyclin (PGI_2_) release from the gut in response to mesenteric traction. The administration of nonsteroidal anti‐inflammatory drugs (NSAID) inhibits PGI_2_ release, stabilizing the hemodynamic response. Here, we examined the effect of mesenteric traction on splanchnic blood flow in pigs randomized to NSAID or placebo treatment.

**Materials and Methods:**

Twenty pigs were allocated to either ketorolac or placebo treatment. Five minutes of manual mesenteric traction was applied. Plasma 6‐keto‐PGF1_α_, a stable metabolite of PGI_2_, hemodynamic variables, and regional blood flow (laser speckle contrast imaging) to the liver, stomach, small intestine, upper lip, and snout (laser Doppler flowmetry) were recorded prior to traction and 5 and 30 minutes thereafter.

**Results:**

Both groups of pigs presented a decrease in systemic vascular resistance (*P* = .01), mean arterial blood pressure (*P* = .001), and blood flow in the gastric antrum (*P* = .002). Plasma 6‐keto‐PGF1_α_ did not increase in either group (*P* = .195), and cardiac output, heart rate, central venous pressure, and blood flow to the liver, small intestine, upper lip, and snout remained unchanged.

**Conclusion:**

Mesenteric traction resulted in cardiovascular depression, including reduced blood flow in the gastric antrum. Plasma 6‐keto‐PGF1_α_ did not increase, and ketorolac administration did not alter the response to mesenteric traction. Furthers studies are needed to identify which substance is responsible for eliciting the cardiovascular response to mesenteric traction in pigs.

## 1 INTRODUCTION

Mesenteric traction syndrome (MTS) is induced by traction and manipulation of the bowel and mesentery during open abdominal surgery,^1^, ^2^ and an incidence of up to 80% is reported.^2^, ^3^, ^4^ The syndrome is considered to be elicited by the vasodilator prostacyclin (PGI_2_) released by endothelial cells upon stimulation, and a triad of hypotension, tachycardia, and facial flushing is provoked. The hemodynamic response is sudden, often presenting within 5 minutes of traction, and the severity of the PGI_2_‐induced hypotension is usually moderate,^1^, ^5^ but severe and prolonged hypotension with little effect of vasopressor treatment has been described.^1^, ^6^ Also, severe postoperative complications after esophagectomy, gastrectomy, and pancreatic resection have been associated with MTS.^2^, ^7^

Splanchnic tissue blood flow is sensitive to hypotension,^8^, ^9^ and postoperative gastrointestinal tract complications are likely associated with inadequate blood flow to splanchnic organs during surgery.^10^, ^11^, ^12^ Thus, hypotension in response to MTS could influence splanchnic tissue blood flow, but evaluation of this association is lacking. To attenuate the MTS response, treatment with nonsteroidal anti‐inflammatory drugs (NSAID) is efficient, and both preoperative and intraoperative administrations of NSAID stabilize the hemodynamic response to MTS.^1^, ^4^, ^13^

The anatomic organization and neurochemical and electrophysiological features of the autonomic nervous system in pigs are considered homologous to humans,^14^ and, therefore, the study was conducted in pigs. We randomized pigs to ketorolac or placebo intervention for evaluation of MTS following mesenteric traction. We evaluated plasma PGI_2_, hemodynamic variables, and splanchnic and facial skin blood flow by laser speckle contrast imaging (LSCI) and hypothesized that ketorolac would inhibit the PGI_2_ release in response to mesenteric traction and thus maintain cardiovascular integrity.

## 2 MATERIALS AND METHODS

This single‐blinded, randomized controlled trial was approved by the Danish Animal Experiments Inspectorate (2014‐15‐0201‐00385) in accordance with the Danish and the European Union Directive 2010/63/EU for animal experiments, and the manuscript adheres to the Animal Research: Reporting of In Vivo Experiments (ARRIVE) guidelines. Twenty female pigs (Danish Landrace/Yorkshire, 10‐12 weeks of age) with a mean weight of 41.9 kg (SD ± 2.4) were included. The animals were housed in pairs and acclimatized for 2 weeks at the Department of Experimental Medicine (The Panum Institute, University of Copenhagen, DK) under standardized room temperature, humidity, and light‐dark cycles. No standardized feeding regime was applied, but the last feeding was provided >12 hours before anesthesia. The primary outcome was splanchnic blood flow assessed by LSCI, with hemodynamic variables and plasma PGI_2_ as secondary outcomes.

### 2.1 Randomization

Twenty pigs were allocated in a 1:1 ratio to the administration of either 10 mg intravenous ketorolac (ketorolac group) or saline (placebo group), and randomization (www.random.org) was concealed by numbered envelopes padded with nontransparent paper. A veterinarian was responsible for the envelopes and attested adherence to the randomization protocol by initiating the administration of ketorolac/placebo while the principal investigator was blinded during the study.

### 2.2 Animal instrumentation

As per standard care at the Department of Experimental Medicine (The Panum Institute, University of Copenhagen, Denmark), the pigs were anesthetized by propofol (15 mg/kg/h) and fentanyl (5 µg/kg/h). A pulmonary artery catheter was introduced via the right external jugular vein for the determination of cardiac output (CO) by thermodilution (Vigilance Monitor, Edwards Life Sciences, Irvine, CA, USA). For continuous monitoring of blood pressure, a catheter was inserted in the right femoral artery, and hemodynamic variables were recorded by PowerLab 16/35 (AD Instruments, Dunedin, NZ) with recordings of CO, mean arterial pressure (MAP), and heart rate (HR). Systemic vascular resistance (SVR) was $\text{SVR}=\frac{80\times \left(\text{MAP}‐\text{CVP}\right)}{\text{CO}}$. Blood samples for plasma PGI_2_ were drawn from a catheter in the left femoral artery.

### 2.3 Blood flow measurements

Blood flow was assessed by laser speckle contrast imaging (LSCI; MoorFLPI, Moor Instruments Ltd., Axminster, UK) placed parallel to the surface of the tissue at a distance of 25 cm.^15^ By LSCI, blood flow was obtained in real time without tissue contact.^15^ Regions of interest (ROIs) were marked on the liver (segment 3), the stomach (antrum: 3 cm from the pylorus, and corpus: 3 cm from the greater curvature), the small intestine (10 cm from the cecum), and the upper lip. Also, blood flow was assessed on the snout by laser Doppler flowmetry (LDF)^16^ chosen because, here, the prone position of the pig prohibited perpendicular positioning of the LSCI camera. Blood flow in the selected ROIs was determined post hoc by a programmed algorithm^15^ (Python versus 2.7.6, Python Software Foundation, Wilmington, USA). All measurements (LSCI/LDF) during the study were obtained at the same ROIs and represent recordings over 30 seconds.

### 2.4 Plasma PGI_2_

Plasma 6‐keto‐PGF_1α_, a stable metabolite of PGI_2_, was chosen for determination because of its prolonged plasma half‐life (approximately 30 minutes^17^) and measured by a commercially available enzyme‐linked immunosorbent assay (ELISA) kit (ADI‐900‐00, Enzo Life Science, Lörrach, DE).

### 2.5 Experimental protocol

Hemodynamic variables and plasma 6‐keto‐PGF_1α_ were obtained after induction of anesthesia. Then, a midline laparotomy was carried out, exposing the abdominal organs to mark ROIs on the organs. After 15 minutes of stabilization, 10 mg intravenous ketorolac (ketorolac group) or saline (placebo group) was administered. After an additional 45 minutes of stabilization (60 minutes after laparotomy, “baseline”), to allow for the effect of the drug, regional blood flow, hemodynamic variables, and plasma 6‐keto‐PGF_1α_ were measured. A 45‐minute stabilization was chosen, as NSAID administration at 15, 90, or 120 minutes before mesenteric traction has proven prophylactic against MTS whereas administration just before surgery has not.^4^ Following stabilization, continuous traction on the stomach and duodenum was applied for 5 minutes with 2 hands by the same individual as described by Brinkmann et al^1^ Measurements of regional blood flow, hemodynamic variables, and plasma 6‐keto‐PGF_1α_ were subsequently collected 5 and 30 minutes after applied traction. These intervals were chosen because the hemodynamic response to mesenteric traction is prompt,^1^ and in prospective, randomized, placebo‐controlled trials (NSAID versus placebo), the response was restored (or no longer statistically different) in both groups within 30 minutes.^1^, ^18^

### 2.6 Statistics

The administration of NSAID has been shown to almost completely inhibit the PGI_2_ release in response to bowel manipulation in humans.^4^ Hence, assuming that MTS does not occur in pigs treated by ketorolac, 20 animals were considered to be required to detect a difference in plasma PGI_2_ after mesenteric traction (*α*: 0.05, and power: 0.85). The statistical analysis was performed with SPSS (Version 22.0., IBM, Armonk, NY, USA) and graphs constructed by GraphPad Prism software (Version 7.0, San Diego, CA, USA). To test for differences between groups at a single time point, a Mann‐Whitney *U* test was applied. The endpoints were entered as the dependent variable in a linear mixed effect model, and Box‐Cox transformed to reduce variance and to achieve assumptions required by the modeling approach. In the analyses, pig individual identification was entered as a random effect to correct for pseudoreplications. To control for false discovery rate, the Benjamini‐Hochberg method was employed.^19^ Data are presented as median with interquartile range, and a *P*‐value <.05 was considered statistically significant.

## 3 RESULTS

Twenty pigs were included for analysis, and the randomization was balanced (ketorolac n = 10; placebo n = 10), and body weight was similar in the 2 groups (41.9 kg (SD ± 2.4); *P* = .684).

A significant interaction between time and placebo showed that placebo had an effect.

### 3.1 Intentional traction

**FIGURE 1:**
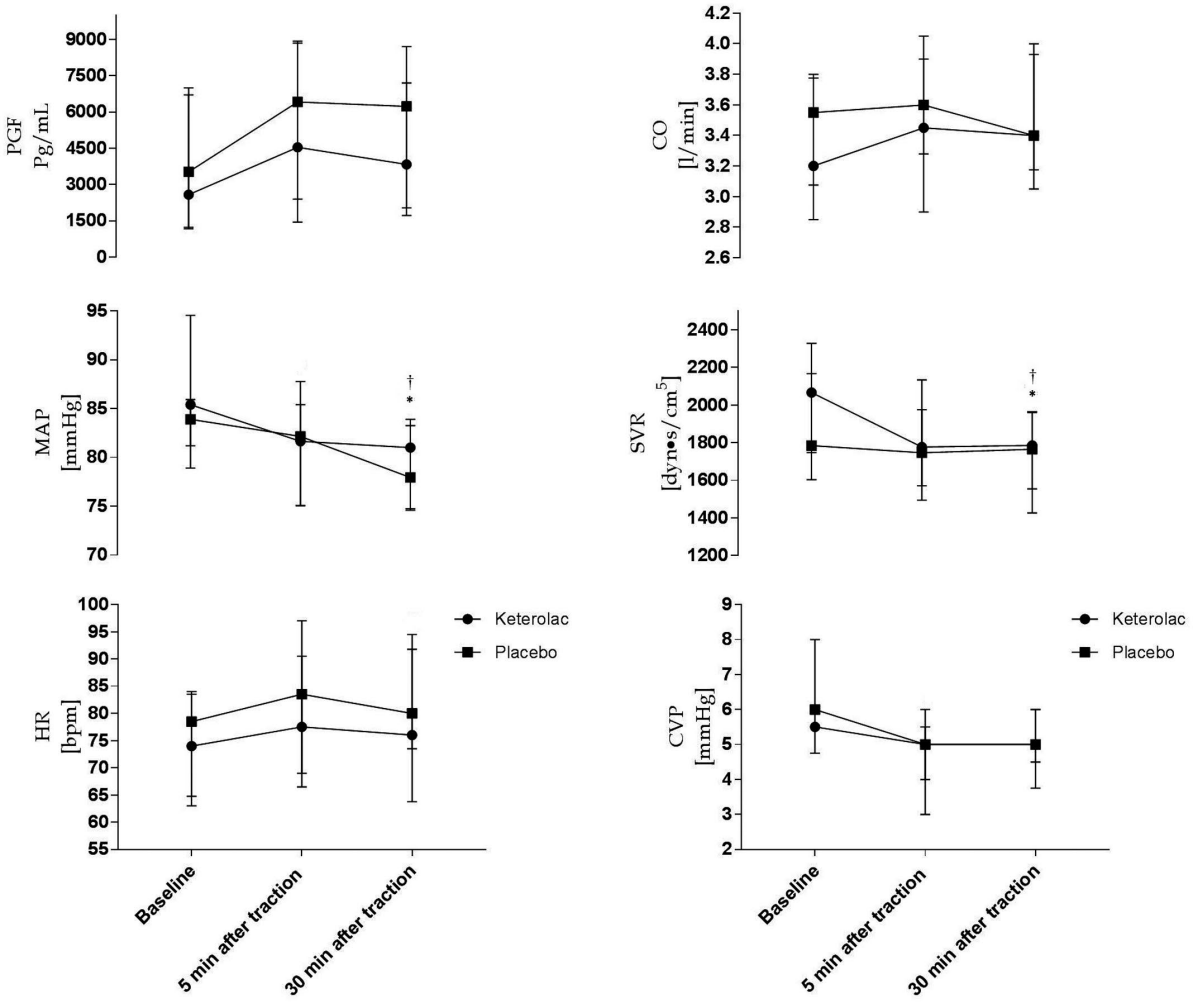
Plasma 6‐keto‐PGF_1α_ and hemodynamic variables in response to intentional traction. Different from baseline in the ketorolac (*) or the placebo (^†^) group, *P* < .05

**FIGURE 2:**
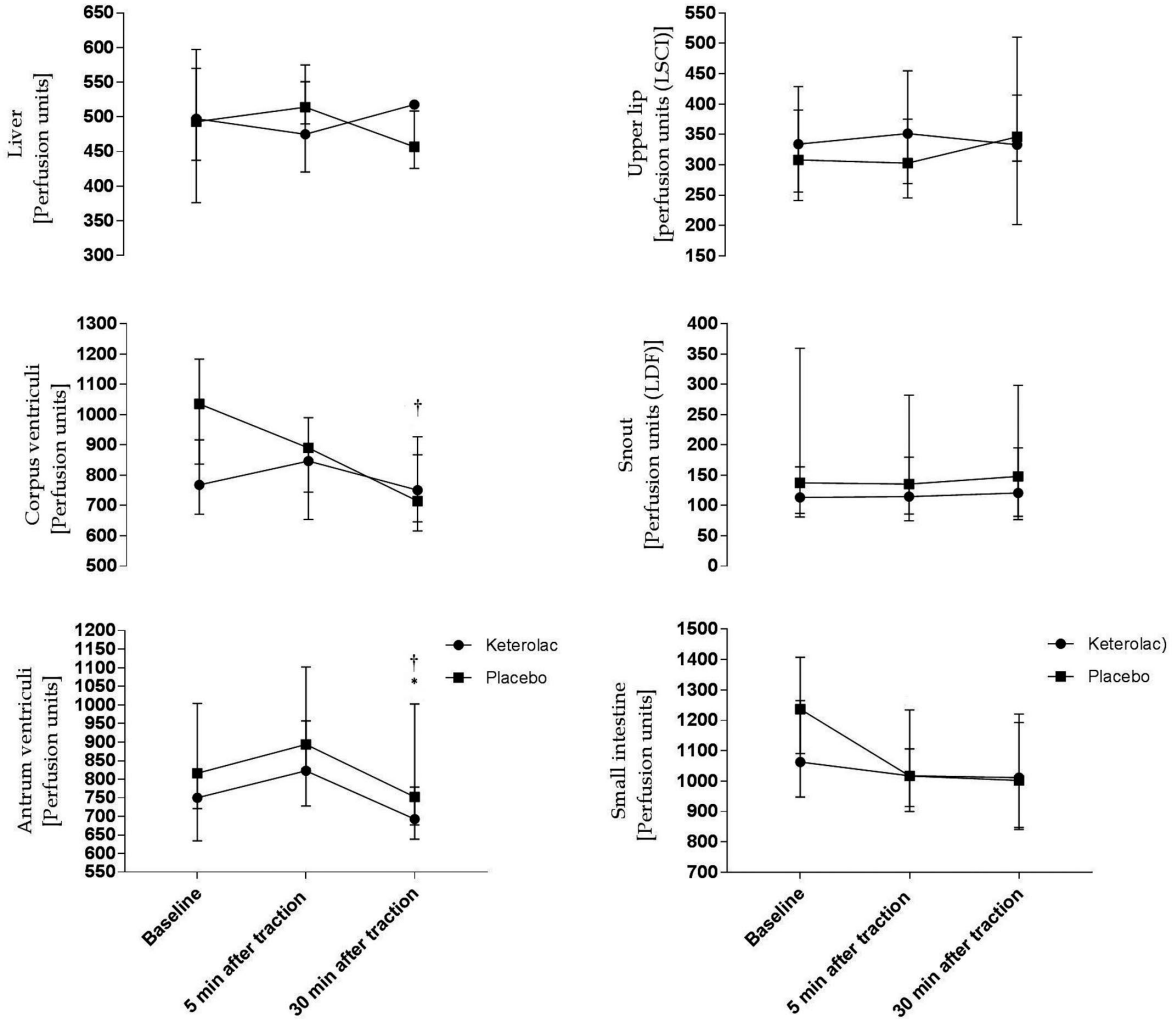
Changes in blood flow in response to intentional traction. Different from baseline in the ketorolac (*) or the placebo (^†^) group, *P* < .05

Blood flow measurements before and after abdominal traction are presented in Figure 1 and Table 1, and plasma 6‐keto‐PGF_1α_, along with hemodynamic variables, is presented in Figure 2 and Table 1. Values were stable at baseline apart from blood flow to the gastric corpus, which was elevated in the placebo group (*P* = .019). In both groups of pigs, SVR, MAP, and blood flow to the gastric antrum decreased after traction (SVR *P* = .001, MAP *P* = .0009, gastric antrum *P* = .002) with no difference between groups (SVR *P* = .692, MAP *P* = .585, gastric antrum *P* = .222). Blood flow to the liver, small bowel, and snout remained stable in both groups. When examining the blood flow to the gastric corpus, a significant interaction between the groups was observed (*P* = .002), where blood flow remained stable in the ketorolac group and decreased in the placebo group. No changes in plasma 6‐keto‐PGF_1α_ after mesenteric traction (*P* = .195) were found, nor were there any changes in HR, CVP, or CO in either of the 2 groups.

**TABLE 1:** Plasma 6‐keto‐PGF_1α_, hemodynamic variables, and blood flow after mesenteric traction presented as median (IQR)

		Baseline 60 min after laparotomy	5 min after traction	30 min after traction
6‐keto‐PGF_1α_ (pg/mL)	Ketorolac	2590 (1238‐6715)	4544 (2407‐8857)	3939 (2040‐7202)
	Placebo	3526 (1168‐7007)	6422 (1457‐8936)	6239 (1727‐8711)
		*P* = *.796*		
MAP (mm/Hg)	Ketorolac	85 (79‐95)	82 (75‐88)	81 (75‐84)*
	Placebo	84 (81‐86)	82 (75‐85)	78 (75‐83)^†^
		*P* = *.481*		
HR (bpm)	Ketorolac	74 (63‐84)	78 (67‐97)	76 (64‐95)
	Placebo	79 (65‐84)	84 (69‐91)	80 (74‐92)
		*P* = *.436*		
CO (L/min)	Ketorolac	3.2 (3.1‐3.8)	3.5 (3.3‐3.9)	3.4 (3.1‐3.9)
	Placebo	3.6 (2.9‐3.8)	3.6 (2.9‐4.1)	3.4 (3.2‐4.0)
		*P* = *.631*		
SVR (dyn∙s/cm^5^)	Ketorolac	2066 (1747‐2166)	1777 (1571‐1975)	1786 (1555‐1960)*
	Placebo	1784 (1603‐2327)	1747 (1494‐2134)	1765 (1427‐1964)^†^
		*P* = *.661*		
CVP (mm/Hg)	Ketorolac	6 (5‐8)	5 (3‐6)^†^	5 (4‐6)
	Placebo	6 (5‐6)	5 (4‐6)	5 (5‐6)
		*P* = *.661*		
LSCI antrum (perfusion units)	Ketorolac	750 (634‐819)	823 (728‐957)	693 (638‐779)*
	Placebo	816 (721‐1004)	894 (829‐1102)	752 (630‐844)^†^
		*P* = *.165*		
LSCI corpus (perfusion units)	Ketorolac	768 (672‐917)	847 (654‐884)	751 (647‐927)
	Placebo	1036 (837‐1183)	890 (744‐990)	715 (616‐868)^†^
		*P* = .019		
LSCI liver (perfusion units)	Ketorolac	497 (376‐570)	475 (420‐575)	518 (384‐546)
	Placebo	493 (437‐597)	514 (490‐551)	457 (426‐508)
		*P* = *.529*		
LSCI small intestine (perfusion units)	Ketorolac	1063 (947‐1265)	1018 (917‐1106)	1012 (841‐1193)
	Placebo	1237 (1091‐1408)	1017 (900‐1235)	1002 (848‐1221)
		*P* = *.089*		
LSCI upper lip (perfusion units)	Ketorolac	334 (241‐429)	352 (245‐455)	333 (202‐510)
	Placebo	308 (255‐390)	303 (269‐375)	346 (306‐415)
		*P* = *.853*		
LDF snout (perfusion units)	Ketorolac	114 (87‐164)	115 (75‐180)	121 (77‐195)
	Placebo	138 (81‐360)	135 (86‐282)	148 (82‐299)
		*P* = *.579*		

Note

Different from baseline (60 minutes after laparotomy) in the ketorolac‐group (*) or placebo group (^†^), *P* < .05.

## 4 DISCUSSION

The principal findings were significant reductions in SVR, MAP, and blood flow to the stomach in both the placebo and the ketorolac group after intentional mesenteric traction and manipulation of the stomach. However, traction did not alter blood flow to the small intestine or liver, nor did plasma 6‐keto‐PGF_1α_ change in either of the 2 groups.

In patients, traction on the abdominal content provokes MTS in 72%‐100% of cases.^1^, ^4^, ^13^ Data on the effects of mesenteric traction in pigs are limited, and to our knowledge, it has not been investigated previously. In humans, the release of vasodilating prostacyclin (PGI_2_) by endothelial cells into the vascular bed upon traction is considered to be the primary causative agent of the syndrome.^1^, ^5^ Prostacyclin exerts vasodilatory effects also in pigs; the intravascular infusion of PGI_2_ decreases splanchnic vascular resistance and thereby lowers blood pressure.^20^ Hence, if mesenteric traction would cause PGI_2_ release also in pigs, we would expect cardiovascular depression. However, mesenteric traction did not increase the plasma concentration of 6‐keto‐PGF_1α_ (a stable metabolite of PGI_2_) in either of the 2 groups of pigs; thus, the decreases in MAP, SVR, and blood flow to the stomach observed in both groups must presumably be driven by factors other than 6‐keto‐PGF_1α_. As mentioned, PGI_2_ is considered the mediator behind MTS. Yet, mast cell‐derived vasoactive mediators could play a role.^21^ Histamine release from mast cells of the small intestine in response to manipulation initiates the triad of hypotension, tachycardia, and facial flushing that characterizes MTS.^21^ In a surgical setting, these symptoms were reduced by the administration of antihistamines.^21^ In pigs, histamine decreases splanchnic vascular resistance, but it is unknown whether manual manipulations of the intestine promote its release.^22^ Numerous vasodilatory substances could be involved in MTS, and their role could differ between humans and pigs.^21^, ^23^ For example, intravenous administration of substance P has little effect on splanchnic circulation in humans, whereas, in pigs, it is a potent vasodilator and increases splanchnic blood flow.^23^

Perioperative administration of NSAID has a prophylactic effect against MTS, and patients who develop MTS present 7 to 20 times larger increases in plasma 6‐keto‐PGF_1α_ than patients treated with NSAID.^1^, ^4^, ^13^ Ketorolac is a potent inhibitor of PGI_2_ production and thereby hampers its vasodilatory effect and elicits potent analgesic, anti‐inflammatory, and antipyretic actions in both pigs and humans.^24^ However, administration of ketorolac did not affect the plasma concentration of 6‐keto‐PGF_1α_ after mesenteric traction. Indomethacin (NSAID) increases vascular resistance and blood pressure in pigs, attributed to the inhibition of PGI_2_ production.^20^ As such, NSAID has the same vasoactive effect in humans and pigs.^20^ In humans, the recommended dosage of ketorolac is 10 mg every 6 hours,^25^ and we therefore considered that the administered dose of ketorolac (10 mg) in this study should have been adequate for inhibiting the plasma PGI_2_ response after mesenteric traction. However, pigs may require a higher dose of ketorolac than do humans, and hence the dose may have been too low to affect PGI_2_ production.^24^ Also, the plasma concentration was not determined.

In contrast to the reduction of blood flow to the stomach, blood flow to the small intestine and liver remained unchanged after mesenteric traction. The mesenteric vascular anatomy in pigs differs from that of humans and other animals.^26^ Primarily the branching pattern of cranial mesenteric vessels gives rise to small arteries radiating towards the mesojejunum, which could cause high resistance to flow. However, it is not clear what effect this vascular organization has on the regulation of blood flow. In contrast, the arteries to the stomach and duodenum follow the arrangement in humans and other mammals.^26^ This discrepancy in mesenteric vascular anatomy could perhaps explain why we did not observe a reduction in blood flow to the small intestine, but only to the stomach.

Blood flow to the gastric antrum was reduced in both groups, and interestingly, flow to the gastric corpus was only reduced in the placebo group (*P* = .002), perhaps indicating some protective effect of ketorolac. As presented in the results section, there was a significant difference in corpus blood flow at baseline (*P* = .019); however, this cannot explain the difference in blood flow over time as this is considered in the statistical model. Also, the coinciding reduction of blood flow to the antrum (*P* = .002) and stable levels of plasma 6‐keto‐PGF_1α_ in both groups argue against ketorolac being the only influencing factor. The sample size calculation of the study was, perhaps optimistically, based on the assumption that NSAID would reduce the level of plasma 6‐keto‐PGF_1α_ to the same extent in pigs as in patients. We did not experience any significant changes in plasma 6‐keto‐PGF_1α_ in either group, and thus, we could be underpowered with regards to other endpoints.

Other limitations should be considered. Mesenteric traction was conducted as described by Brinkman et al^1^ However, the traction was not quantified, and the methodology of traction could have been standardized by using a pully with weights or similar techniques.

LSCI, rather than LDF, was chosen to evaluate splanchnic blood flow because the device does not require tissue contact, thus avoiding compression of capillaries in the region of interest and enabling the assessment of blood flow in a large tissue area.^15^ Furthermore, LSCI has excellent reproducibility when evaluating microperfusion in the skin^27^ and splanchnic organs.^15^

Abdominal exploration and mesenteric traction reduced blood flow to the stomach, possibly because of both loss of SVR and a decrease in MAP. Nevertheless, plasma 6‐keto‐PGF_1α_ remained unchanged, and the administration of ketorolac did not influence the hemodynamic variables in response to mesenteric traction. Hence, hemodynamic instability upon mesenteric traction may be attributed to mediators other than plasma PGI_2,_ and further studies are needed to identify the mechanisms behind the hemodynamic response to mesenteric traction in pigs.

## CONFLICT OF INTEREST

The authors report no proprietary or commercial interest in any product mentioned or concept discussed in this article.

## AUTHOR CONTRIBUTIONS

R. Strandby, J. Osterkamp, R. Ambrus, M. Achiam, and L. B. Svendsen have all made substantial contributions to the concept and design of the work. R. Strandby, J. Osterkamp, R. Ambrus, A. Henriksen, J. P. Goetze, N. H. Secher, M. Achiam, and L. B. Svendsen have made substantial contributions to the acquisition, analysis, and interpretation of the data for this work. R. Strandby, J. Osterkamp drafted the article, and all authors revised it critically for important intellectual content. All authors agree to be accountable for all aspects of the work and ensuring that questions related to the accuracy or integrity of any part of the work are appropriately investigated and resolved. All authors have approved the final version of this paper for publication.
